# Blood Pressure Patterns and Hepatosteatosis: Cardiometabolic Risk Assessment in Dipper and Non-Dipper Phenotypes

**DOI:** 10.3390/jcm13226976

**Published:** 2024-11-19

**Authors:** Ramazan Astan, Dimitrios Patoulias, Ana Ninić, Ramazan Dayanan, Paschalis Karakasis, Tolga Mercantepe, Filiz Mercantepe, Aleksandra Klisic

**Affiliations:** 1 Department of Cardiology, Batman Training and Research Hospital, Batman 72000, Türkiye; ramazan.astan@saglik.gov.tr; 2 Outpatient Department of Cardiometabolic Medicine, Aristotle University of Thessaloniki, General Hospital “Hippokration”, 54124 Thessaloniki, Greece; dipatoulias@gmail.com; 3 Second Department of Internal Medicine, European Interbalkan Medical Center, 57001 Thessaloniki, Greece; 4 Department of Medical Biochemistry, Faculty of Pharmacy, University of Belgrade, 11000 Belgrade, Serbia; aninic@pharmacy.bg.ac.rs; 5 Department of Endocrinology and Metabolism, Batman Training and Research Hospital, Batman 72000, Türkiye; ramazan.dayanan@saglik.gov.tr; 6 Second Department of Cardiology, Aristotle University of Thessaloniki, General Hospital “Hippokration”, 54124 Thessaloniki, Greece; pakar15@hotmail.com; 7 Department of Histology, Faculty of Medicine, Recep Tayyip Erdogan University, Rize 53100, Türkiye; 8 Department of Endocrinology and Metabolism, Faculty of Medicine, Recep Tayyip Erdogan University, Rize 53100, Türkiye; 9 Faculty of Medicine, University of Montenegro, 81000 Podgorica, Montenegro; aleksandranklisic@gmail.com; 10 Center for Laboratory Diagnostics, Primary Health Care Center, 81000 Podgorica, Montenegro

**Keywords:** hypertension, dipper, non-dipper, hepatosteatosis, cardiometabolic risk, uric acid

## Abstract

**Background/Objectives**: Non-dipper hypertension (HT), a condition in which blood pressure does not drop sufficiently at night compared to daytime, is considered a serious condition that increases the risk of cardiovascular disease, stroke, and organ damage. This study aimed to examine the relationship between dipper and non-dipper blood pressure patterns, hepatosteatosis, and biochemical markers in hypertensive and normotensive individuals. **Methods:** Demographic, biochemical, and hepatic ultrasonography data from 142 patients who underwent 24 h ambulatory blood pressure measurement (ABPM) were evaluated retrospectively and cross-sectionally in this study. Patients were categorized into four groups based on ABPM results: non-dipper normotensive (NDN), dipper normotensive (DN), non-dipper hypertensive (NDH), and dipper hypertensive (DH). **Results:** The study results indicate that NDH individuals had markedly elevated levels of hepatosteatosis and uric acid compared with DH and normotensive persons (*p* < 0.001). The grade of hepatosteatosis showed significant discriminatory capacity in differentiating between dipper and non-dipper hypertensive patients, with an AUC of 0.861, specificity of 94%, and sensitivity of 66%. Individuals with hypertension exhibiting a non-dipper pattern demonstrate a greater prevalence of hepatosteatosis and elevated uric acid levels. **Conclusions:** The study findings show non-dipper patterns have a higher risk for cardiometabolic diseases. This indicates that not only blood pressure, but also metabolic disorders should be closely monitored and treated in the management of non-dipper HT.

## 1. Introduction

Hypertension (HT) is a prevalent cardiovascular risk factor globally [1]. Inadequate treatment of hypertension patients can lead to severe consequences, including coronary artery disease, cerebrovascular illness, and chronic renal disease [2,3]. HT should be evaluated not only by office blood pressure or daytime measurements, but also via a 24 h blood pressure profile [4]. The circadian rhythm of blood pressure is a physiological characteristic that significantly influences the development and prognosis of cardiovascular illnesses. The circadian rhythm pattern of HT can be elucidated using 24 h ambulatory blood pressure measures (ABPM), which is the endorsed gold standard for monitoring this cycle [5]. In healthy individuals, blood pressure is often anticipated to fall by 10–20% during nocturnal hours [6]. The reduction is referred to as the “dipper” phenotype, serving as a preventive attribute for cardiovascular health. This decrease permits the heart to experience diminished pressure during the night, yielding beneficial effects on the vascular system and lowering the incidence of cardiovascular incidents. The lack of or reduction in this nocturnal decline, referred to as the “non-dipper” pattern in certain individuals, is linked to a heightened risk of metabolic and cardiovascular disease [7]. The non-dipper pattern may result in detrimental vascular consequences, including arterial stiffening, endothelial dysfunction, left ventricular hypertrophy, and heightened sympathetic nervous system activity [7,8]. This circumstance also influences metabolic processes, leading to metabolic disorders such as insulin resistance and dyslipidemia [9]. Nondipping is believed to affect glucose and lipid metabolism, hence elevating the risk of metabolic syndrome and diabetes [10]. In this perspective, using circadian blood pressure patterns in hypertension treatment provides a holistic strategy that can enhance both blood pressure regulation and metabolic and vascular health.

Hepatosteatosis is a metabolic condition that causes an excessive buildup of fat in the liver and is frequently associated with metabolic syndrome [11,12]. Hypertension, a significant element of metabolic syndrome, can induce hepatosteatosis, and the simultaneous presence of both diseases can exacerbate cardiometabolic risk [13,14]. Furthermore, limited information suggests that the prevalence of hepatosteatosis is elevated in non-dipper hypertensive people [13]. A correlation exists between fatty liver and circadian rhythm, similar to the association between alterations in circadian rhythm and hypertension patterns [15,16]. In this context, examining metabolic disparities between non-dipper and dipper phenotypes is crucial for informing clinical management.

This study involved 142 patients who conducted 24 h ambulatory blood pressure measures. The patients were categorized into four groups: non-dipper normotensive, dipper normotensive, non-dipper hypertension, and dipper hypertensive. The groups were compared regarding demographics, biochemical parameters, and hepatosteatosis. Our objective in conducting this comparison is to enhance our understanding of the cardiometabolic risk factors potentially linked to hepatosteatosis, particularly by elucidating the distinctions between non-dipper and dipper individuals. Considering that the relationship between non-dipper phenotype and hepatosteatosis and biochemical parameters has been examined in a limited number of studies in the literature, we believe that this study can fill the gap in knowledge in this area.

## 2. Participants and Methods

This retrospective study included data from 142 patients who performed 24 h ambulatory blood pressure monitoring at the Cardiology Department of Batman Training and Research Hospital from January 2021 to March 2022. Approval for the study was received from the Local Ethics Committee of Batman Training and Research Hospital (Approval Number: 75144452-929-3483, (270)/2021, Approval date: 23 March 2021). The research was carried out in compliance with the ethical standards of the Declaration of Helsinki.

### 2.1. Study Design and Data Collections

This retrospective cross-sectional study utilized electronic health records to gather demographic data, biochemical parameters, and hepatosteatosis status. In instances of incomplete information, patients were contacted through telephone numbers recorded in the hospital system to obtain the missing data. The requisite sample size for the ANOVA test in the study was determined to be a minimum of 144, divided into four groups, with 85% power, 0.05 type I error rate, and a 0.3 effect size, as per G power analysis. The study initially included 144 participants based on power analysis; however, due to the inability to obtain demographic data on two individuals via the hospital’s computerized registration system or via telephone, these two individuals were eliminated from the study.

In 24 h ABPM, patients exhibiting daytime systolic blood pressure (SBP) ≥ 135 mm Hg and/or diastolic blood pressure (DBP) ≥ 85 mm Hg, overnight SBP ≥ 120 mm Hg and/or DBP ≥ 70 mm Hg, or a 24 h mean SBP ≥ 130 mm Hg and/or DBP ≥ 80 mm Hg were categorized as hypertensive. Individuals who failed to satisfy these criteria were categorized as normotensive [17]. Measurements for 24 h ABPM were conducted with commercially certified, noninvasive equipment (Rozinn RZ250 ABP recorder, SN R 02157/0807, Glendale, NY, USA). Measurements were conducted at 20 min intervals throughout the day (06:00–22:00) and at 30 min intervals during the night (22:00–06:00). The cuff was positioned on the patient’s non-dominant arm. All patients were directed to maintain their usual activities during ABPM. Patients’ periods of sleep and wakefulness were documented based on their self-reported information. Computer software was employed to examine the recordings. Patients who had a failure rate exceeding 20% in their blood pressure measures were eliminated from the trial. The 24 h ABPM mean for each patient was determined using the hourly averages of daytime and overnight systolic and diastolic blood pressure. Patients were categorized as having dipper HT if their nightly SBP and DBP means were at least 10% lower than their daytime means, and as having non-dipper HT if the reduction was less than 10% [18]. In this study, patients with acute and chronic liver failure, acute and chronic renal failure, secondary hypertension, cerebrovascular diseases, acute and chronic infections, connective tissue diseases, autoimmune diseases, hematological disorders, malignancies, and steroid users were excluded due to their potential influence on hypertension and blood pressure patterns, which could confound the study’s results. Liver failure can alter vascular tone by affecting plasma protein equilibrium and electrolyte concentrations [19]. The kidneys regulate blood pressure via the renin-angiotensin-aldosterone system (RAAS); thus, reduced RAAS activity in persons with renal failure may result in hypertension [20]. Furthermore, renal failure can influence uric acid and electrolyte equilibrium, resulting in alterations in blood pressure patterns [20]. In secondary hypertension, assessing blood pressure patterns characteristic of initial hypertension becomes challenging [21]. Alterations in sympathetic nervous system activity resulting from cerebrovascular disorders may influence blood pressure patterns [22]. Infections can alter vascular resistance, either elevating or diminishing it, via inflammatory cytokines, resulting in hypertension or hypotension [23]. Nonetheless, this is typically transient and may hinder the precise study of hypertension trends. Connective tissue disorders and autoimmune diseases influence inflammation and vascular tone, thereby impairing blood pressure regulation [23]. Certain connective tissue disorders, particularly vasculitis, can directly impact the vascular architecture and alter the pattern of hypertension [24]. Hematological diseases can influence vascular tone and blood pressure by altering blood viscosity and oxygen-carrying capacity [25]. Hypotension may occur in anemia, but hypertension can arise in situations such as polycythemia [25]. Chemotherapy and radiotherapy employed in malignancy treatment exert diverse impacts on the circulatory system, potentially elevating or diminishing blood pressure [26]. Furthermore, inflammation, coagulation abnormalities, and metabolic alterations in cancer patients may result in disturbances in blood pressure patterns [26]. Steroids, particularly with prolonged usage, can elevate blood pressure by inducing fluid and sodium retention, in addition to altering blood pressure patterns [27]. All these factors were eliminated from the study to more precisely evaluate the effects related to the internal dynamics of primary hypertension.

The individuals’ body weight was measured using a medical scale and height with a calibrated meter, employing established methods to compute BMI. Weight measurements were conducted in the morning on an empty stomach, without shoes, wearing a single layer of clothing, and with an empty bladder. The body mass index (BMI) was computed with the formula BMI = [(weight in kilograms)/(height in meters)^2^] [28,29]. The obesity definition was established at a BMI threshold value ≥ 30 kg/m^2^ [30,31]. All anthropometric measures were conducted using a consistent approach by the same qualified healthcare expert.

The hepatosteatosis score was assessed using liver ultrasound (Samsung RS85 GB 2022, Chuncheon, Republic of Korea). Ultrasonography has been employed for the objective evaluation of hepatic lipid buildup and hepatic injury. Liver ultrasounds were conducted on all study participants in the morning following a 12 h fast, performed by the same radiologist who was blinded to the study groups. The degrees of hepatosteatosis were determined using the echo differences of the liver and kidney [32]. Normal liver parenchyma generally presents on ultrasound as a homogenous tissue with echogenicity comparable to or somewhat above that of the renal cortex and spleen. Fatty infiltration of the liver may result in altered echogenicity [32,33]. Due to hepatosteatosis, the liver exhibits increased echogenicity on ultrasonography compared to the renal cortex and spleen, where it appears bright or hyperechoic. A prevalent grading system categorizes steatosis into grades 0, I, II, and III. The grades are determined by the level of echogenicity and the extent to which specific landmarks are obscured. Grade 0 steatosis indicates normal hepatic architecture devoid of fat buildup in the liver. Grade I steatosis signifies a small elevation in echogenicity, with the liver exhibiting a marginally brighter appearance compared to the renal cortex and spleen. Grade II steatosis signifies a considerable elevation in echogenicity, resulting in a brighter liver and the obscuration of the echogenic walls of the portal vein branches. Grade III steatosis indicates a substantial elevation in echogenicity, rendering the liver sufficiently bright to conceal the shape of the diaphragm [32,33].

All study participants underwent a single antecubital venous blood draw following a 12 h fasting period. Two tubes were subjected to centrifugation at 4000× *g* for 10 min to isolate their serum. Routine biochemical parameters including fasting blood glucose, uric acid, alanine aminotransferase (ALT), aspartate aminotransferase (AST), total cholesterol (TC), triglycerides (TG), HDL-cholesterol (HDL-C), LDL-cholesterol (LDL-C), C-reactive protein (CRP), and hormone levels were analyzed in the Biochemistry Laboratory of Batman Education and Research Hospital. The hexokinase technique was employed to quantify glucose levels. Total cholesterol, triglycerides, HDL-C, and LDL-C levels were quantified using the photometric method via the autoanalyzer (Abbott Architect c16000, Abbot Park, Chicago, IL, USA). A complete blood count was conducted utilizing the Sysmex XN-1000 (Sysmex Co., Kobe, Japan) automated hematological cell counter. Nephelometric analysis was employed to identify CRP (Siemens Healthcare Diagnostics Products GmbH, 35041 Marburg, Germany, Type BN II System, SN: 202826). The chemiluminescence spectrophotometric technique (Beckman Coulter, Brea, CA, USA) was employed to quantify the following biochemical parameters: sodium, potassium, AST, ALT, and uric acid.

### 2.2. Study Groups

**Figure 1:**
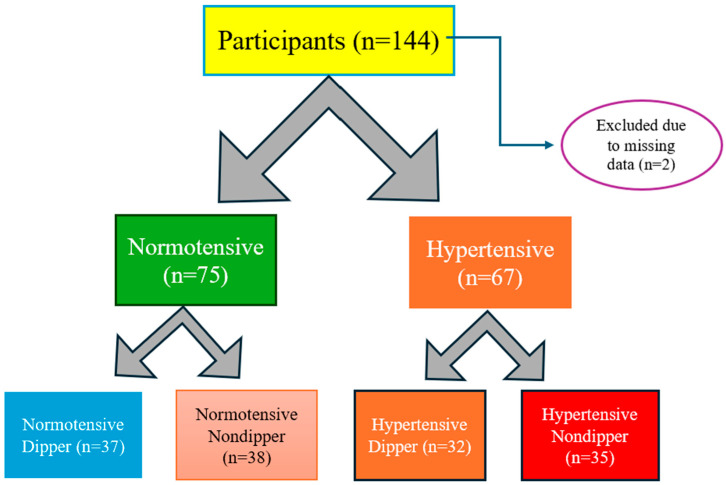
Schematic presentation of the study design.

In the first step, 142 participants of the study were categorized into two groups: hypertension and non-hypertensive. Demographic, clinical, and biochemical characteristics were compared between the two groups. The flow chart showing the design of the study is presented in Figure 1.

In the second step, participants were divided into four groups based on blood pressure measurements and dipper characteristics:

- Dipper normotensive (ND), (*n* = 37): Participants who are normotensive have a normal drop in blood pressure at night.
- Non-dipper normotensive (NND), (*n* = 38): Participants who are normotensive as a result of 24 h ABPM and whose blood pressure does not drop sufficiently at night.
- Dipper hypertensive (HD), (*n* = 32): Participants who are hypertensive and have a normal drop in blood pressure at night.
- Non-dipper hypertensive (HND), (*n* = 35): Participants who are hypertensive and whose blood pressure does not drop sufficiently at night.

### 2.3. Statistical Analysis

The distribution of the data was tested using the Shapiro–Wilk test. The results are given as the arithmetic mean (standard deviation) for normally distributed variables and as the median (interquartile range) for skewed variables. Differences in continuous variables between the groups studied were tested using the one-way ANOVA with Tukey post-hoc test for normally distributed variables and the Kruskal–Wallis with the Mann–Whitney post hoc test for skewed variables. The Chi-square test for contingency tables was used to test for differences in categorical variables. The association between the variables and the presence of non-dipper hypertension was examined using univariate and multivariate binary logistic regression analysis. Adjustment in the multivariable analysis was performed for all continuous variables that were statistically associated with non-dipper hypertension and for categorical variables that differed significantly between the tested groups. Variables in the logistic regression analysis were presented as odds ratio (OR) and 95% confidence interval (CI). Receiver operating characteristic (ROC) curve analysis was used to distinguish between dipper and non-dipper patients and between dipper and non-dipper hypertensive patients. The variables of the ROC analysis were presented as areas under the ROC curve (AUC) and 95% CI. At an AUC between 0.7 and 0.8, the diagnostic test has satisfactory accuracy, at an AUC between 0.8 and 0.9, the diagnostic test has good accuracy, and at an AUC above 0.9, the diagnostic test has excellent accuracy. A *p* value of less than 0.05 was considered statistically significant. All statistical calculations were performed with SPSS^®^ Statistical package version 21 (Chicago, IL, USA).

## 3. Results

The study commenced with a comparative analysis of the data from normotensive and hypertensive groups. Comparison of demographic and clinical data of normotensive (NT) and hypertensive (HT) groups is presented in Table 1. There were more men in the HT group than in the NT group. They were also older than the NT. As expected, all SBD and DBP levels measured in this study were significantly higher in the HT than in the NT. HT groups were more likely to take calcium channel blockers, ACE inhibitors, aldosterone receptor blockers, thiazide diuretics, and oral antidiabetics than NT groups. There were more smokers and patients with type 2 diabetes among the HT group. There were no differences in the degree steatosis between the two tested groups.

Table 2 shows laboratory comparisons between NT and HT groups. Glucose concentration, uric acid levels, and vitamin D concentration were higher in HT than in NT. Conversely, ALT activity, hemoglobin, and B12 concentrations were lower in HT than in NT.

In the second step, the normotensive and hypertensive cohorts were categorized into two groups based on dipper and non-dipper patterns, and data from the four distinct subgroups were compared. The clinical characteristics of the subgroups are listed in Table 3. There were more male patients in the NND, HD, and HND groups than in the ND group. HND patients were older than patients in the ND and NND groups. SBD daytime, SBP nighttime, SBP average, DBP daytime, DBP nighttime, and DBP average were significantly higher in HD and HND than in normotensive patients. SBP nighttime was also higher in HND than in HD. Compared to the other three groups, HND patients most frequently took calcium channel blockers, ACE inhibitors, aldosterone receptor blockers, and thiazide diuretics. However, HND patients took more beta-blockers than ND and NND patients. HD patients took more ACE inhibitors and thiazide diuretics than ND and NND patients. There were more smokers and patients with type 2 diabetes among the hypertensive patients. Hypertension patients took more oral antidiabetics than ND patients. There were more patients with a higher degree of steatosis among the HND patients than among the ND and HD patients. There were also more patients with a higher degree of steatosis in HD than in ND and NND groups.

Table 4 presents comparative laboratory analyses of the four classifications. The glucose concentration was significantly higher in HD patients than in ND and NND patients (Table 2). Uric acid levels were highest in HND patients. In addition, HD patients had higher uric acid levels than ND patients. Only NND patients had higher uric acid levels than ND patients. Total cholesterol and TG were lower in HND patients than in HD patients. ND patients had higher TG levels than NND and HND patients. However, TG levels were higher in HD patients than in NND patients. There were no significant differences between the other examined biochemical markers (Table 2).

Associations between clinical and biochemical markers and non-dipper status were examined in all patients using univariable binary regression analysis (Table 5). The independent variable was dichotomous, indicated as 0—dipper status and 1—non-dipper status.

Steatosis grade, heart rate, uric acid, total cholesterol, and TG showed significant OR in the univariable logistic regression. As steatosis grade rose for 1 stadium, heart rate for 1 beat, and uric acid for 1 mg/dL, the odds of non-dippers increased by 4.6-fold, 4.3%, and 1.74-fold, respectively. The risk of non-dippers was 1.5% and 0.4% higher when total cholesterol and TG concentrations decreased by 1 mg/dL, respectively.

Nagelkerke R^2^ showed that steatosis grade, heart rate, uric acid, total cholesterol, and TG could explain 38.1%, 4.7%, 11.7%, 7.3%, and 9.0% of the variation in the development of non-dipper tension, respectively.

In addition, a multivariable logistic regression analysis was performed to determine markers independently associated with the risk of developing non-dipper tension in the population studied (Table 3). All predictors tested in the univariable analysis that were significantly associated with non-dipper status were tested in the multivariable analysis. The model was adjusted for all antihypertensive and oral antidiabetic therapies that differed significantly between the groups studied (Table 1). In multivariate logistic regression analysis, steatosis grade, uric acid, and TG were found to be independent predictors of the development of non-dipper tension (OR = 4.383, *p* < 0.001, OR = 1.976, *p* = 0.008, and OR = 0.994, *p* = 0.004, respectively). According to Nagelkerke R^2^, the model was able to explain 57.6% of the variation in non-dipper development.

**Figure 2:**
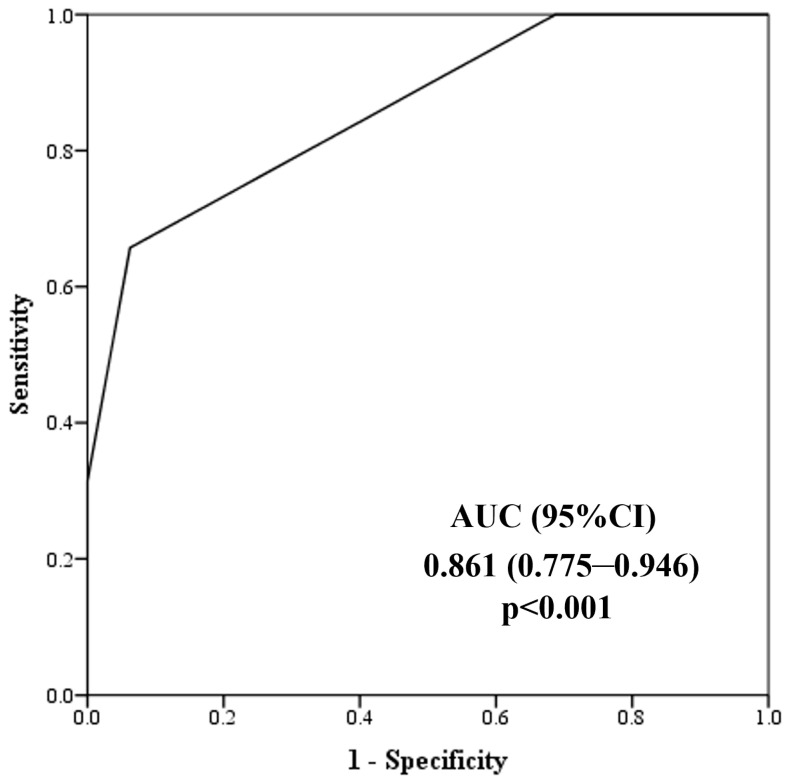
Discriminatory power of steatosis grade towards dipper and non-dipper in all patients.

To determine the potential utility of a single marker to discriminate non-dippers from dippers in the entire study population, a ROC analysis was performed (Figure 2). The AUC for steatosis grade was 0.797 with 95% CI (0.726–0.869). The discriminatory ability of the steatosis grade indicates satisfactory accuracy (AUC between 0.7 and 0.8). The steatosis grade had a specificity of 84% and a sensitivity of 58% to detect non-dippers in the study group.

The investigated markers, which showed a significant OR for the prediction of non-dipper status in all patients in the univariable binary regression analysis, were further investigated for possible associations with non-dipper status, but only in hypertensive patients (Table 6). Steatosis grade and uric acid were significantly positively associated with non-dipper status in hypertensive patients, as well as in all patients. Total cholesterol and TG were also significantly negatively associated with non-dipper status in hypertensive patients, as in all patients. As steatosis grade rose and uric acid for 1 mg/dL the odds of hypertensive non-dippers increased by 15.4-fold, and 1.5-fold, respectively. The risk of non-dippers was 2.1% and 0.7% higher when total cholesterol and TG concentrations decreased by 1 mg/dL, respectively.

Nagelkerke R^2^ showed that steatosis grade, uric acid, total cholesterol, and TG could explain 57.5%, 9.7%, 13.7%, and 17.9% of the variation in the development of non-dipper hypertension, respectively.

In addition, a multivariable logistic regression analysis was performed to determine markers independently associated with the risk of developing non-dipper hypertension (Table 5). All predictors tested in the univariable analysis that were significantly associated with non-dipper status were tested in the multivariable analysis. The model was adjusted for all antihypertensive therapy significantly different between HD and HND patients (Table 1). In multivariate logistic regression analysis, steatosis grade and TG were found to be independent predictors of the development of non-dipper hypertension (OR = 18.383, *p* = 0.008 and OR = 0.990, *p* = 0.046, respectively). According to Nagelkerke R^2^, the model was able to explain 80.9% of the variation in non-dipper development.

**Figure 3:**
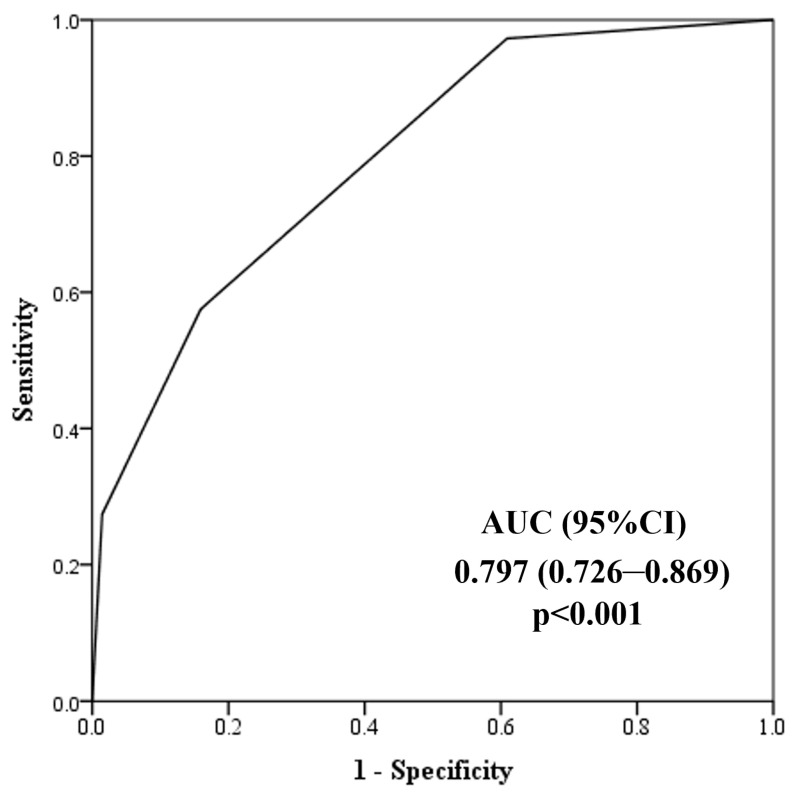
Discriminatory power of steatosis grade towards hypertensive dipper and non-dipper patients.

An ROC analysis was performed to determine the potential utility of steatosis grade to differentiate between hypertensive non-dippers and dippers (Figure 3). The AUC for steatosis grade was 0.861 with 95% CI (0.775–0.946). The discriminatory power of the steatosis grade indicates good accuracy (AUC between 0.8 and 0.9). The steatosis grade had a specificity of 94% and a sensitivity of 66% to detect non-dippers in hypertensive patients.

## 4. Discussion

The present study revealed significant differences in demographics, biochemicals, and hepatosteatosis between normotensive and hypertensive patients with non-dipper and dipper blood pressure patterns. The findings of our study show that non-dipper hypertension pattern, which carries a higher cardiometabolic risk, is closely associated with hepatosteatosis and biochemical disorders. The study findings confirm that hypertensive patients are older than normotensive patients and that the male gender is dominant.

Nevertheless, NDH patients were older than DH patients and had a higher proportion of males. The prevalence of smoking and type 2 diabetes was elevated among hypertension individuals. This situation underscores the necessity of meticulously managing not only blood pressure, but also metabolic and lifestyle parameters in persons with hypertension. The elevated frequency of type 2 diabetes among hypertensive individuals highlights the possible correlation between insulin resistance and fatty liver in this demographic. Furthermore, our results substantiate the correlation between hypertension and male gender, age, diabetes, and smoking, while demonstrating that age and gender serve as risk factors for both hypertension and the non-dipper pattern.

The study results indicate that the prevalence of hepatosteatosis was markedly elevated in non-dipper hypertensive patients. This finding is supported by the literature [13]. Notably, there was no distinction in hepatosteatosis between the normotensive and hypertension cohorts. This may suggest that the blood pressure pattern exerts a more significant influence on hepatosteatosis than hypertension. The non-dipper blood pressure pattern is more closely linked to effects such as damage to target organs and stiff arteries. The fact that hepatosteatosis risk is included shows that these people have a bigger problem [34]. In addition, non-dipper hypertension is also more frequently associated with other components of the metabolic syndrome [35]. This finding indicates that the cardiometabolic risk for this patient group is significantly elevated. 

Our analysis revealed that the extent of hepatosteatosis possesses a significant ability to differentiate non-dipper hypertensive individuals. This finding indicates that hepatosteatosis could serve as a significant predictor in non-dipper hypertensive individuals. Prior research indicated a bidirectional association between hepatosteatosis and hypertension; specifically, hypertension may induce hepatosteatosis, while hepatosteatosis may exacerbate the severity of hypertension [36]. To comprehend the correlation between hepatosteatosis and non-dipper hypertension, it is essential to identify the shared etiopathogenetic variables that contribute to both clinical conditions. Age, obesity, metabolic syndrome, insulin resistance, diabetes mellitus, catecholamines, renin, aldosterone, cortisol, melatonin, abnormal neurohormonal regulation, which is characterized by an imbalance between sympathetic and parasympathetic nervous system tone, insufficient physical activity, irregular eating patterns, increased sodium consumption, and tobacco use are some extrinsic and intrinsic factors that contribute to the development of a non-dipper blood pressure [9,10,37,38,39,40,41,42,43,44,45,46,47,48,49]. Furthermore, many of these factors are also risk factors for hepatosteatosis [12]. Considering each of these components independently will facilitate the assessment of the impacts of inflammation, oxidative stress, and hormonal abnormalities on the patterns of hepatosteatosis and non-dipper hypertension. Hepatosteatosis is defined by the buildup of lipids and metabolic impairment in the liver, potentially resulting in systemic inflammation [50]. In persons with fatty livers, elevated pro-inflammatory cytokines (e.g., TNF-α, IL-6) compromise arterial endothelial function, hence heightening the risk of hypertension [50]. Non-dipper hypertension is linked to heightened sympathetic nervous system activity and less parasympathetic activity, potentially exacerbating inflammation and oxidative stress levels [10]. Oxidative stress is a significant component that reinforces the connection between these two disorders. Hepatosteatosis and non-dipper hypertension are both marked by elevated reactive oxygen species and insufficient antioxidant defense mechanisms. Fatty liver may compromise endothelial function by elevating oxidative stress levels, adversely impacting blood pressure regulation, and potentially inducing a nondipping pattern. Furthermore, oxidative stress may elevate vascular stiffness, rendering blood pressure more resistant. Hormonal imbalances also influence the connection between these two illnesses. Insulin resistance significantly contributes to the pathogenesis of hepatosteatosis and non-dipper hypertension; elevated cortisol levels may accompany increased insulin resistance, potentially resulting in alterations in blood pressure patterns [40,51]. Elevated stress hormones, particularly cortisol, are prevalent in persons with hepatosteatosis, and these hormones may exacerbate blood pressure levels [10,47,52,53]. Cortisol may disturb circadian rhythms, especially in non-dipper hypertensive patients, inhibiting nocturnal blood pressure lowering. Thus, the overlapping pathophysiological mechanisms of the two disorders necessitate combined attention in the clinical therapy of patients.

The study confirms the correlation between hypertension and uric acid levels, demonstrating markedly elevated serum uric acid levels in non-dipper individuals. The impact of uric acid levels on renal function must also be evaluated in relation to non-dipper hypertension. While uric acid has a beneficial function at low concentrations owing to its antioxidant characteristics, elevated levels indicate heightened oxidative stress and may result in endothelial dysfunction and vascular impairment [34,53,54,55,56]. The accumulation of uric acid in the kidneys may result in renal impairment, hence aggravating hypertension [53]. This condition leads to heightened renal stress owing to the elevated nocturnal blood pressure observed in non-dipper individuals. Elevated uric acid levels in non-dipper persons may heighten the risk of renal failure, with this correlation being more significant in comparison to dipper individuals [51]. Moreover, an alternative explanation for the disparity in uric acid levels between dipper and non-dipper patterns is the heightened activity of the sympathetic nervous system in non-dipper individuals [51,53]. This augmentation may influence oxidative stress levels, resulting in elevated uric acid concentrations. Consequently, additional examination of the relationship between uric acid and oxidative stress in non-dipper hypertensive persons may enhance comprehension of vascular and renal health hazards in this population.

Interestingly, the biochemical tests revealed that total cholesterol and triglyceride levels were significantly lower in non-dipper hypertensive individuals than in the dipper group. LDL levels were reduced in non-dipper hypertensive patients; however, the change was not statistically significant. This may suggest insufficient metabolic regulation in patient groups exhibiting a dipper pattern or an increased utilization of triglyceride-lowering nonpharmacological agents in NDH patients. Moreover, patients with HT exhibited a greater frequency of DM. Consequently, they may exhibit greater adaptability to lifestyle modifications. The utilization of both insulin and oral antidiabetic medications in this group may also elucidate this circumstance. Insulin is a hormone that significantly influences lipid metabolism in the body. It can modulate triglyceride and total cholesterol levels via many pathways [57]. Initially, insulin stimulates the lipoprotein lipase (LPL) enzyme, facilitating the hydrolysis of triglycerides and their transformation into free fatty acids [58]. The absorption of triglycerides into muscle and adipose tissue rises, resulting in a reduction in triglyceride levels in plasma [58]. Concurrently, insulin inhibits the synthesis of very low-density lipoprotein (VLDL) in the liver [59]. The inhibition of VLDL particles, which are abundant in triglycerides, aids in lowering circulating triglyceride levels [59]. Insulin inhibits lipolysis in adipose tissue, hence diminishing the flow of free fatty acids into the bloodstream [59]. This action aids in regulating triglyceride levels by inhibiting the accumulation of triglycerides in plasma. Insulin stimulates HMG-CoA reductase, a crucial enzyme influencing cholesterol synthesis in the liver [60]. This may elevate intracellular cholesterol levels, prompting the liver to diminish the absorption of LDL from plasma. Insulin reduces plasma LDL cholesterol levels via enhancing LDL receptor expression in hepatic cells [57,59]. Consequently, insulin exerts an indirect influence on cholesterol levels. Non-insulin antidiabetic medications exert varying effects on triglycerides and cholesterol levels [59]. The medications that confer the most substantial advantages on the lipid profile are metformin, GLP-1 agonists, and pioglitazone; other drugs exhibit neutral or minimal effects on lipid levels [59]. Metformin effectively reduces triglyceride levels by inhibiting gluconeogenesis in the liver and enhancing cellular energy expenditure [61]. Furthermore, it diminishes lipogenesis by enhancing adenosine monophosphate kinase (AMPK) activity and aids in reducing plasma triglyceride levels by suppressing VLDL formation [62]. Metformin may also yield a reduction in total cholesterol and LDL cholesterol levels. Research indicates that GLP-1 agonists, which enhance insulin secretion and diminish glucagon release through GLP-1 receptors, may effectively lower LDL cholesterol and triglyceride levels while elevating HDL cholesterol levels [63]. Thiazolidinediones enhance insulin sensitivity by activating peroxisome proliferator-activated receptor gamma (PPAR-γ), leading to reduced lipolysis in adipose tissue and the liver, hence suppressing VLDL generation and elevating HDL levels [64,65]. A further explanation may stem from an inadequate sample size. Nonetheless, a less known issue is that LDL cholesterol functions as a negative acute phase reactant [66]. While non-dipper hypertension cannot be classified as an acute inflammatory condition and CRP levels do not corroborate this, the hypothesis that LDL may function as an acute phase reactant in subclinical inflammatory states should pave the way for extensive future research. In conclusion, the elevated hepatosteatosis in non-dipper hypertensive patients indicates that dyslipidemia in these individuals demonstrates distinct biochemical patterns and that hepatosteatosis may develop through mechanisms separate from those of dyslipidemia.

The findings of the current investigation reveal another intriguing result. No difference in CRP levels was observed between the study groups. Literature suggests that HT and cardiovascular diseases are inflammatory conditions [67,68,69]. Observational studies and meta-analyses in the literature highlight a rather constant bidirectional association between HT and elevated CRP levels [66,70,71]. CRP is a nonspecific and sensitive acute-phase reactant detectable in the initial stages of diseases [70,72]. Due to the rapid increase in circulating CRP levels following an inflammatory response, it has historically been used as a biomarker for systemic inflammation [73]. Research indicates that elevated CRP levels are present in HT and may potentially serve as a predictor of future HT in normotensive individuals [72]. Elevated CRP levels in people with HT typically signify inadequate blood pressure regulation or the onset of hypertensive problems, and CRP is frequently elevated in individuals with high baseline blood pressure, encompassing both systolic and diastolic measurements [66]. This concept is founded on the premise that elevated CRP levels enhance angiotensin receptor expression, stimulate the production of plasminogen activator inhibitor-1 (PAI-1), activate vascular smooth muscle, induce the release of inflammatory mediators, diminish the responsiveness of vascular endothelial cells to vasodilatory agents, decrease nitric oxide synthesis, and elevate vascular resistance. Simultaneously, during an inflammatory reaction, CRP is primarily synthesized by the liver in response to interleukin-1 (IL-1), interleukin-6 (IL-6), and tumor necrosis factor-alpha (TNF-α) [72]. CRP is typically higher in hepatosteatosis, indicating that the formation of hepatic adipose tissue correlates with heightened inflammation-induced oxidative stress [72]. There could be other explanations for the lack of difference in CRP levels between the groups in the study described. Primarily, nearly all studies in literature employed the high sensitivity-CRP (hs-CRP) test, which can precisely quantify CRP levels below 10.0 mg/L to assess low-grade inflammation [66,70]. Nonetheless, hs-CRP was not examined in our research. This may have resulted in inadequate sensitivity to identify low-grade inflammation in HT. A second factor is because, inherently, most hypertension patients were undergoing diverse antihypertensive therapies. Furthermore, diabetes mellitus was more prevalent among these individuals, who were receiving antidiabetic therapy. Medications for hypertension, dyslipidemia, and diabetes management may have mitigated the rise in CRP levels by reducing inflammation [66]. This scenario has also been documented in the literature [66]. Nevertheless, our sample size precluded the execution of subgroup analyses to ascertain the potential effects of each category of medicines.

Antihypertensive medications influence CRP levels [66]. Angiotensin II possesses proinflammatory characteristics. Angiotensin II receptor blockers demonstrated the ability to lower CRP levels independently of their impact on blood pressure in randomized studies including hypertensive individuals. Likewise, it has been demonstrated that the administration of renin–angiotensin–aldosterone system inhibitors or beta blockers as monotherapy correlates with reduced CRP levels in comparison to diuretic usage [66]. Nonetheless, it is unclear whether these medications influence CRP levels independently of their impact on blood pressure. In the presented study, the rate of using antidiabetic treatment was higher in HT patients. Oral antidiabetic medications, especially SGLT2 inhibitors, recognized for their antihypertensive properties, recently garnered attention for their potential antioxidant, anti-inflammatory, and immunomodulatory actions [74,75]. Moreover, comprehensive research, particularly the JUPITER study, demonstrated that statin medications lower CRP levels independently of their impact on cholesterol concentrations [76,77]. The beneficial effects of statins may be facilitated by their ability to counteract CRP-induced endothelial dysfunction and oxidative stress. The reduction in CRP levels due to statin therapy is recognized as a significant marker of treatment efficacy, with advantages comparable to those achieved by lowering LDL cholesterol levels [66,72].

The study’s concluding observation indicated that blood pressure patterns did not significantly influence vitamin D levels; nonetheless, hypertensive patients exhibited elevated amounts of D vitamin. We believe there is just one explanation for this. Individuals with hypertension were inherently subjected to greater medical oversight and received replacement therapy more frequently than those with normal blood pressure. The reduced hemoglobin observed in the hypertensive cohort can be attributed to the use of prophylactic antiplatelet treatment, necessitated by the elevated cardiovascular disease risk associated with both hypertension and diabetes mellitus, which consequently may lead to occult bleeding.

This study indicates that non-dipper hypertension individuals exhibit a greater cardiometabolic risk than dipper hypertensive patients, with a notable prevalence of hepatosteatosis being a significant concern in this group. Our research emphasizes the necessity for enhanced cardiovascular risk management in non-dipper hypertensive individuals and recommends that metabolic abnormalities, such as hepatosteatosis, be assessed more routinely in this population. The therapeutic significance of identifying non-dipper hypertension is crucial for both cardiovascular risk and metabolic and organ functions [36]. The non-dipper pattern may exacerbate metabolic disorders, particularly inflammation, insulin resistance, and endothelial dysfunction, potentially imposing additional stress on the liver, resulting in dysfunction, and heightening the risk of developing metabolic syndrome components, such as fatty liver [9]. Consistent assessment of liver function tests in non-dipper hypertensive patients enhances the formulation of a more effective monitoring and treatment approach, given their elevated risk profile. This surveillance is particularly crucial for people predisposed to liver disorders or those with concurrent metabolic syndrome. Clinicians should routinely evaluate liver function and account for metabolic risk factors, alongside managing blood pressure, in patients with non-dipper hypertension to enhance overall patient health.

Nonetheless, certain strengths and limitations of our study must be taken into account throughout its evaluation. The primary advantage of our study is its design utilizing 24 h ABPM, regarded as the gold standard for identifying dipper and non-dipper patterns. The adequate quantity of participants allowed us to conduct comparisons among the groups. Furthermore, our study analyzed both blood pressure changes and biochemical and metabolic parameters. The limitations of our study include its single-center and retrospective design and insufficient data regarding participants’ lifestyle characteristics and dietary habits. The gender disparity among participants in our study limited our ability to compare males and females. Moreover, ultrasonography, a technique characterized by inadequate sensitivity and specificity, was employed instead of biopsy, which is the gold standard for evaluating hepatosteatosis. Some participants used antihypertensive, antidyslipidemic, and antidiabetic medications. The impact of these drugs on blood pressure patterns and metabolic indicators was not entirely regulated; maybe this situation is constraining the generalizability of the findings.

## 5. Conclusions

The current study revealed that non-dipper hypertension individuals had elevated levels of hepatosteatosis and uric acid compared to dipper hypertensive and normotensive patients, suggesting an increased cardiometabolic risk in this cohort. The heightened incidence of hepatosteatosis in non-dipper hypertensive individuals suggests that fatty liver disease warrants closer surveillance in this demographic. Non-dipper hypertension necessitates more intensive treatment options for managing cardiovascular and metabolic risks. These findings highlight that the early diagnosis of non-dipper hypertension in clinical practice should encompass not only blood pressure management, but also the treatment of metabolic and biochemical abnormalities. Future prospective studies may help us understand how non-dipper hypertension affects long-term cardiometabolic outcomes and devise new ways to treat this group of patients.

In conclusion, non-dipper hypertension is a significant factor in both the treatment of hypertension and the management of cardiometabolic risks. Formulating individualized treatment methods through interdisciplinary communication for this patient cohort will enhance long-term outcomes.

## Figures and Tables

**Table 1:** Demographic and clinical characteristics are for normotensive and hypertensive groups.

	Normotensive Group (NT) N = 75	Hypertensive Group (HT) N = 67	*p*
Male, N (%) *	16 (21)	31 (46)	0.002
Age, years	52.7 ± 13.2	60.7 ± 12.3	<0.001
BMI, kg/m^2^	27.0 ± 1.7	27.0 ± 1.6	0.936
SBP daytime, mmHg †	121 (114–127)	142 (129–150)	<0.001
SBP nighttime, mmHg †	108 (103–117)	130 (120–139)	<0.001
SBP average, mmHg †	119 (112–125)	140 (127–146)	<0.001
DBP daytime, mmHg	75 ± 9	88 ± 11	<0.001
DBP nighttime, mmHg	67 ± 8	78 ± 12	<0.001
DBP average, mmHg	74 ± 9	85 ± 11	<0.001
Heart rate †	80 (69–84)	80 (72–84)	0.953
Calcium channel blockers, N (%) *	0 (0)	14 (21)	<0.001
ACE inhibitors, N (%) *	0 (0)	22 (33)	<0.001
Aldosterone receptors blockers, N (%) *	0 (0)	14 (21)	<0.001
Beta blockers, N (%) *	0 (0)	9 (13)	0.001
Thiazide diuretics, N (%) *	0 (0)	28 (42)	<0.001
Smokers, N (%) *	14 (19)	28 (42)	0.003
Hyperlipidemia, N (%) *	14 (19)	17 (25)	0.334
Statins, N (%) *	0 (0)	2 (3)	0.132
Type 2 diabetes, N (%) *	7 (9)	25 (37)	<0.001
Oral antidiabetics, N (%) *	6 (8)	21 (31)	<0.001
Insulin, N (%) *	1 (1)	3 (4)	0.258
Oral antidiabetics + insulin, N (%) *	1 (1)	3 (3)	0.258
Steatosis grade, N (%) *			0.367
0	19 (25)	10 (15)	
1	28 (37)	32 (48)	
2	18 (24)	14 (21)	
3	10 (13)	11 (16)	

Data are presented as mean ± SD and compared by Student *t*-test. † Data are presented as median (interquartile range) and compared by Mann–Whitney test. * Data are presented as absolute and relative frequencies and compared by Chi-square test for contingency tables.

**Table 2:** Comparisons of biochemical markers between normotensive and hypertensive groups.

	Normotensive Group (NT), N = 75	Hypertensive Group (HT), N = 67	*p*
Glucose, mg/dL	95 (90–108)	109 (93–148)	0.005
Uric acid, mg/dL	4.2 (4.0–4.7)	4.8 (4.0–6.1)	0.001
CRP, mg/L	0.40 (0.14–0.70)	0.41 (0.20–0.85)	0.511
Total cholesterol, mg/dL	180 (162–197)	180 (157–200)	0.765
HDL-C, mg/dL †	45.6 ± 9.1	46.0 ± 10.2	0.820
LDL-C, mg/dL	112 (94–121)	108 (85–124)	0.186
TG, mg/dL	130 (98–188)	150 (109–195)	0.177
AST, IU/L	21 (16–26)	18 (15–22)	0.068
ALT, IU/L	19 (14–26)	17 (13–21)	0.035
Na, mEq/L	140 (139–142)	140 (138–142)	0.853
K, mEq/L	4.4 (3.9–4.6)	4.2 (4.1–4.7)	0.463
WBC	7.40 (6.66–8.60)	7.55 (6.70–8.44)	0.974
Neutrophils count	4.30 (3.70–5.29)	4.40 (3.62–5.20)	0.802
Lymphocytes count	2.30 (1.90–2.70)	2.40 (1.57–2.64)	0.233
Monocytes count	0.45 (0.37–0.50)	0.45 (0.32–0.58)	0.909
Hemoglobin, g/dL	13.95 (13.10–14.60)	12.10 (11.10–14.40)	0.024
Platelet count	268 (228–310)	269 (244–313)	0.945
Vitamin D, ng/mL	15.27 (7.55–29.65)	34.60 (11.60–46.30)	0.042
Ferritin, ng/mL	49.87 (35.00–66.00)	42.60 (17.00–56.20)	0.204
Vitamin B12, pg/mL	344 (274–425)	323 (267–374)	0.038
Folic acid, ng/mL	8.25 (6.75–9.60)	6.80 (6.10–7.90)	0.138

Data are presented as median (interquartile range) and compared by Mann–Whitney test. † Data are presented as mean ± SD and compared by Student *t*-test.

**Table 3:** Demographic and clinical characteristics of the population studied.

	Normotensive Dipper (ND) N = 37	Normotensive Non-Dipper (NND) N = 38	Hypertensive Dipper (HD) N = 32	Hypertensive Non-Dipper (HND) N = 35	*p*
Male, N (%) ^¶^	4 (11)	12 (32) a#	14 (44) a*	17 (49) a‡	0.003
Age, years	53.0 ± 15.2	52.5 ± 11.1	58.7 ± 13.0	62.6 ± 11.5 a#,b*	0.002
BMI, kg/m^2^	26.8 ± 1.8	27.1 ± 1.5	26.7 ± 1.6	27.2 ± 1.7	0.532
SBP daytime, mmHg †	123 (116–127)	119 (106–125) a‡	145 (141–150) a‡,b‡	125 (95–149) a‡,b‡	<0.001
SBP nighttime, mmHg †	106 (103–113)	112 (105–123)	125 (117–130 a‡,b‡	134 (121–125) a‡,b‡,c*	<0.001
SBP average, mmHg †	120 (113–124)	118 (105–126)	142 (137–144) a‡,b‡	138 (124–151) a‡,b‡	<0.001
DBP daytime, mmHg	78 ± 8	72 ± 9	91 ± 9 a‡,b#	84 ± 12 a‡,b‡	<0.001
DBP nighttime, mmHg	66 ± 8	70 ± 8	76 ± 10 a‡,b#	81 ± 12 a#,b‡	<0.001
DBP average, mmHg	76 ± 8	72 ± 9	88 ± 10 a‡,b#	82 ± 12 a‡,b‡	<0.001
Heart rate † (beats/minute)	76 (70–84)	82 (68–88)	76 (70–83)	80 (74–84)	0.276
Calcium channel blockers, N (%) ^¶^	1 (3)	0 (0)	2 (6)	12 (34) a‡,b‡,c*	<0.001
ACE inhibitors, N (%) ^¶^	0 (0)	0 (0)	5 (16) a#,b#	17 (49) a‡,b‡,c*	<0.001
Aldosterone receptors Blockers, N (%) ^¶^	0 (0)	0 (0)	3 (9)	11 (31) a‡,b‡,c#	<0.001
Beta blockers, N (%) ^¶^	0 (0)	0 (0)	2 (6)	7 (20) a*,b*	0.001
Thiazide diuretics, N (%) ^¶^	0 (0)	0 (0)	6 (19) a*,b*	22 (63) a‡,b‡,c‡	<0.001
Smokers, N (%) ^¶^	4 (11)	10 (26)	16 (50) a‡,b#	12 (34) a#	0.004
Hyperlipidemia, N (%) ^¶^	9 (24)	5 (13)	10 (31)	7 (20)	0.315
Statins, N (%) ^¶^	0 (0)	0 (0)	0 (0)	2 (6)	0.102
Type 2 diabetes, N (%)	2 (5)	5 (13)	13 (41) a‡,b*	12 (34) a*,b#	0.001
Oral antidiabetics, N (%) ^¶^	1 (3)	5 (13)	10 (31) a*	11 (31) a*	0.003
Insulin, N (%) ^¶^	1 (3)	0 (0)	0 (0)	3 (9)	0.099
Oral antidiabetics + Insulin, N (%) ^¶^	1 (3)	0 (0)	0 (0)	3 (9)	0.099
Steatosis grade, N (%) ^¶^					<0.001
0	17 (46)	2 (5) a‡	10 (31) a#,b‡	0 (0) a‡,c‡	
1	11 (30)	17 (45)	20 (63)	12 (34)	
2	8 (22)	10 (26)	2 (6)	12 (34)	
3	1 (3)	9 (24)	0 (0)	11 (32)	

Data are presented as mean ± SD and compared by one-way ANOVA with Tukey post hoc test. † Data are presented as median (interquartile range) and compared by Kruskal–Wallis and Mann–Whitney tests dependent on the number examined groups. ^¶^ Data are presented as absolute and relative frequencies and compared by Chi-square test for contingency tables. a—significantly different from normotensive dipper, b—significantly different from normotensive non-dipper, and c—significantly different from hypertensive dipper. * *p* < 0.01; ‡ *p* < 0.001; and # *p* < 0.05.

**Table 4:** Biochemical markers of tested populations.

	Normotensive Dipper (ND) N = 37	Normotensive Non-Dipper (NND) N = 38	Hypertensive Dipper (HD) N = 32	Hypertensive Non-Dipper (HND) N = 35	*p*
Glucose, mg/dL	94 (90–108)	101 (93–108)	120 (101–138) a‡,b*	101 (90–152)	0.004
Uric acid, mg/dL	4.1 (4.0–4.4)	4.7 (4.0–5.0) a*	4.6 (4.0–5.3) a#	5.0 (4.4–6.9) a‡,b#,c#	<0.001
CRP, mg/L	0.37 (0.17–0.60)	0.45 (0.10–0.79)	0.43 (0.12–0.85)	0.40 (0.20–0.90)	0.926
Total cholesterol, mg/dL	184 (160–198)	179 (169–192)	192 (170–205)	161 (142–191) c*	0.034
HDL-C, mg/dL †	44.8 ± 9.7	46.4 ± 8.7	45.9 ± 12.0	46.0 ± 8.5	0.904
LDL-C, mg/dL	112 (96–120)	111 (92–121)	108 (87–118)	92 (76–127)	0.588
TG, mg/dL	159 (124–188)	108 (76–176) a*	178 (132–254) b*	125 (100–166) a#,c*	0.001
AST, IU/L	21 (17–26)	20 (16–26)	19 (16–23)	18 (15–21)	0.228
ALT, IU/L	16 (14–26)	21 (16–39)	17 (13–21)	17 (13–20)	0.079
Na, mEq/L	139 (138–141)	141 (140–143)	1410 (139–142)	140 (137–142)	0.081
K, mEq/L	4.4 (3.9–4.7)	4.4 (4.0–4.6)	4.2 (4.0–4.7)	4.3 (4.1–4.7)	0.883
WBC	7.13 (6.40–8.80)	7.41 (7.10–7.90)	7.40 (6.70–8.00)	7.60 (6.75–8.60)	0.904
Neutrophils count	4.50 (3.70–5.40)	4.11 (3.50–4.60)	4.04 (3.50–4.95)	4.50 (3.75–5.40)	0.271
Lymphocytes count	2.20 (1.68–2.70)	2.53 (1.90–3.06)	2.50 (1.50–2.69)	2.22 (1.68–2.64)	0.300
Monocytes count	0.44 (0.36–0.50)	0.45 (0.38–0.53)	0.40 (0.28–0.53)	0.50 (0.39–0.65)	0.079
Hemoglobin, g/dL	13.50 (12.50–14.50)	13.55 (13.00–14.30)	13.25 (11.10–14.15)	12.80 (12.00–14.65)	0.153
Platelet count	285 (228–324)	265 (223–298)	269 (248–308)	259 (235–325)	0.902
Vitamin D, ng/mL	10.45 (6.20–28.50)	17.72 (9.30–27.95)	25.76 (13.20–30.24)	18.75 (8.70–41.85)	0.136
Ferritin, ng/mL	29.20 (10.10–63.30)	46.37 (38.20–67.30)	46.95 (17.00–68.20)	56.00 (24.70–139.60)	0.120
Vitamin B12, pg/mL	348 (275–425)	364 (294–435)	294 (234–408)	349 (253–372)	0.174
Folic acid, ng/mL	8.23 (5.40–9.60)	8.80 (6.909.60)	6.50 (5.20–6.80)	7.50 (6.70–8.50)	0.180

Data are presented as median (interquartile range) and compared by Kruskal–Wallis or Mann–Whitney tests dependent on the number examined groups. † Data are presented as mean ± SD and compared by one-way ANOVA with Tukey post hoc test. a—significantly different from normontensive dipper, b—significantly different from normotensive non-dipper, and c—significantly different from hypertensive dipper. * *p* < 0.01; ‡ *p* < 0.001; and # *p* < 0.05.

**Table 5:** Association of studied markers with non-dipper status in normotensive and hypertensive patients using univariable binary regression analysis.

Univariable Analysis			
Marker	OR (95% CI)	*p*	R^2^
Gender	0.535 (0.262–1.093)	0.086	0.028
Age, years	1.010 (0.985–1.035)	0.451	0.005
BMI, kg/m^2^	1.161 (0.947–1.425)	0.152	0.020
Steatosis grade	4.598 (2.674–7.907)	<0.001	0.381
Heart rate	1.043 (1.002–1.086)	0.039	0.047
Cigarette smoking	1.057 (0.514–2.174)	0.881	0.000
Diabetes melitus	1.093 (0.497–2.404)	0.825	0.000
Hyperlipidemia	0.518 (0.229–1.168)	0.518	0.024
Glucose,	0.999 (0.992–1.005)	0.724	0.001
Uric acid,	1.740 (1.256–2.410)	0.001	0.117
CRP	0.826 (0.623–1.096)	0.185	0.022
Total cholesterol,	0.985 (0.974–0.996)	0.007	0.073
HDL-C,	1.010 (0.976–1.045)	0.583	0.003
LDL-C,	0.992 (0.980–1.004)	0.173	0.018
TG,	0.996 (0.992–0.999)	0.011	0.090
AST,	0.995 (0.970–1.021)	0.723	0.001
ALT,	0.999 (0.987–1.012)	0.923	0.000
Na	1.031 (0.911–1.167)	0.629	0.002
K	0.952 (0.828–1.095)	0.490	0.007
WBC	1.058 (0.911–1.229)	0.457	0.005
Neutrophils count	1.059 (0.885–1.268)	0.529	0.004
Lymphocytes count	1.157 (0.734–1.826)	0.530	0.004
Hemoglobin	1.031 (0.841–1.263)	0.772	0.001
Platelet count	1.000 (0.995–1.005)	0.969	0.000
Vitamin D	1.009 (0.976–1.044)	0.593	0.005
Ferritin	1.005 (0.998–1.011)	0.177	0.055
Vitamin B12	1.000 (0.997–1.002)	0.743	0.001
Folic acid	1.215 (0.997–1.481)	0.054	0.083
**Multivariable Analysis**			
**Model**	**OR (95% CI)**	***p***	**R^2^**
Steatosis grade	4.383 (2.340–8.208)	<0.001	0.576
Heart rate	1.044 (0.989–1.102)	0.119	
Uric acid,	1.976 (1.192–3.278)	0.008	
Total cholesterol,	0.994 (0.975–1.006)	0.218	
TG	0.994 (0.989–0.998)	0.004	

The model was adjusted for all antihypertensive and oral antidiabetic therapies.

**Table 6:** Association of markers with non-dipper status in hypertensive patients using binary logistic regression analysis.

Univariable Analysis			
Marker	OR (95% CI)	*p*	R^2^
Steatosis grade	15.469 (3.651–65.548)	<0.001	0.575
Heart rate	1.039 (0.981–1.100)	0.192	0.045
Uric acid,	1.549 (1.038–2.313)	0.032	0.097
Total cholesterol,	0.979 (0.962–0.996)	0.014	0.131
TG,	0.993 (0.987–0.999)	0.032	0.179
**Multivariable Analysis**			
**Model**	**OR (95% CI)**	***p***	**R^2^**
Hepatosteatosis grade	18.386 (2.103–160.702)	0.008	0.809
Uric acid,	1.969 (0.800–4.842)	0.140	
Total cholesterol,	0.998 (0.965–1.032)	0.896	
TG,	0.990 (0.980–1.000)	0.046	

Model was adjusted for antihypertensive therapy significantly different between non-dipper and dipper hypertensive patients.

## Data Availability

All data generated or analyzed during this study are included in this article. The data will be available upon reasonable request (contact persons: filiz.mercantepe@saglik.gov.tr).
